# Supplementary material for: Offline replay supports planning in human reinforcement learning
Source: eLife. 2018 Dec 14;7:e32548. doi: 10.7554/eLife.32548 (PMC6303108; doi:10.7554/eLife.32548)
Supplement: Transparent reporting form [file elife-32548-transrepform.pdf]

eLife's transparent reporting form

We encourage authors to provide detailed information *within their submission* to facilitate the interpretation and replication of experiments. Authors can upload supporting documentation to indicate the use of appropriate reporting guidelines for health-related research (see EQUATOR Network), life science research (see the BioSharing Information Resource), or the ARRIVE guidelines for reporting work involving animal research. Where applicable, authors should refer to any relevant reporting standards documents in this form.

If you have any questions, please consult our Journal Policies and/or contact us: editorial@elifesciences.org.

Sample-size estimation

- You should state whether an appropriate sample size was computed when the study was being designed
- You should state the statistical method of sample size computation and any required assumptions
- If no explicit power analysis was used, you should describe how you decided what sample (replicate) size (number) to use

Please outline where this information can be found within the submission (e.g., sections or figure legends), or explain why this information doesn't apply to your submission:

We based the sample size in our within-subject design on previous fMRI studies from our group with comparable design (Doll et al. 2015). This information is included under the Subjects section of Material and Methods in the accompanying submission.
"This sample size, *n=24*, is justified given previous studies in the field with comparable within-participant designs (*5*)."

Replicates

- You should report how often each experiment was performed
- You should include a definition of biological versus technical replication
- The data obtained should be provided and sufficient information should be provided to indicate the number of independent biological and/or technical replicates
- If you encountered any outliers, you should describe how these were handled
- Criteria for exclusion/inclusion of data should be clearly stated
- High-throughput sequence data should be uploaded before submission, with a private link for reviewers provided (these are available from both GEO and ArrayExpress)

Please outline where this information can be found within the submission (e.g., sections or figure legends), or explain why this information doesn't apply to your submission:

eLife Sciences Publications, Ltd is a limited liability non-profit non-stock corporation incorporated in the State of Delaware, USA, with company number 5030732, and is registered in the UK with company number FC030576 and branch number BR015634 at the address 1st Floor, 24 Hills Road, Cambridge CB2 1JP | August 2014

We refined the behavioral design in a series of 22 pilots conducted on mechanical Turk; the final two versions of the task had n=16 and n=24 participants and (pre-)replicated the key behavioral findings in the fMRI version of the task reported in the paper (also n=24). We do not report the mTruk pilots as replications, but under the Model-based Analysis section of Material and Method, page 26 of initial submission, we report that the learning rate was estimated using our final pilot data with the same n as our fMRI study:
"We estimated a learning rate of .7 based on analysis of previous pilot behavioral data (*n=24*) and used this learning rate for all participants."

Statistical reporting

- Statistical analysis methods should be described and justified
- Raw data should be presented in figures whenever informative to do so (typically when N per group is less than 10)
- For each experiment, you should identify the statistical tests used, exact values of N, definitions of center, methods of multiple test correction, and dispersion and precision measures (e.g., mean, median, SD, SEM, confidence intervals; and, for the major substantive results, a measure of effect size (e.g., Pearson's r, Cohen's d)
- Report exact p-values wherever possible alongside the summary statistics and 95% confidence intervals. These should be reported for all key questions and not only when the p-value is less than 0.05.

Please outline where this information can be found within the submission (e.g., sections or figure legends), or explain why this information doesn't apply to your submission:

- A full description and justification of statistical analysis methods appears in the text.
- The experiment consists of >10 participants per condition (*n=24*, within-subject design). Nonetheless, we have used swarm plots superimposed on violin plots for behavioral results (Figure 2 and Supplementary Figure 2), and scatter plots with data distributions along the axes for our main neural-behavioral correlation results (Figure 2 and Supplementary Figure 2) to clearly display the distribution of the data.
- All statistical information has been provided in the results section of the submitted manuscript. Cohen's d (for repeated measures, c.f. Lakens 2013) and Pearson's r are provided for our major results. See under Behavioral and neuroimaging analysis under Material and methods.

"**Effect size** We calculate and report Cohen's *dz* as a measure of effect size for paired t-tests and Cohen's q to compare correlations *(48).*"

- We report exact p-values throughout the results section.

(For large datasets, or papers with a very large number of statistical tests, you may upload a single table file with tests, Ns, etc., with reference to sections in the manuscript.)

Group allocation

- Indicate how samples were allocated into experimental groups (in the case of clinical studies, please specify allocation to treatment method); if randomization was used, please also state if restricted randomization was applied
- Indicate if masking was used during group allocation, data collection and/or data analysis

eLIFE
1st Floor
24 Hills Road
Cambridge CB2 1JP, UK
P 01223 855340
W elifesciences.org
T @elife

Please outline where this information can be found within the submission (e.g., sections or figure legends), or explain why this information doesn't apply to your submission:

- We used a within subject design where all participants were allocated to all conditions. The order of conditions was randomized for each participant. The full information appears in the submitted manuscript, page 6.
- We used full brain and functional region of interest (ROI) analyses. A full description of ROI selection (using separate functional localizer runs) appears in the submitted manuscript, pages 8 and 10.

Additional data files ("source data")

- We encourage you to upload relevant additional data files, such as numerical data that are represented as a graph in a figure, or as a summary table
- Where provided, these should be in the most useful format, and they can be uploaded as "Source data" files linked to a main figure or table
- Include model definition files including the full list of parameters used
- Include code used for data analysis (e.g., R, MatLab)
- Avoid stating that data files are "available upon request"

Please indicate the figures or tables for which source data files have been provided:

We will release the data on a publicly accessible repository upon publication, but not before. Upon publication, we will provide a more detailed edit to the paper to indicate for which figures and tables the data and analysis codes are available.

eLife Sciences Publications, Ltd is a limited liability non-profit non-stock corporation incorporated in the State of Delaware, USA, with company number 5030732, and is registered in the UK with company number FC030576 and branch number BR015634 at the address 1st Floor, 24 Hills Road, Cambridge CB2 1JP | August 2014
